# Was the European oil industry prepared for the current global crisis?

**DOI:** 10.1007/s13202-022-01529-7

**Published:** 2022-06-30

**Authors:** Romeo Victor Ionescu, Monica Laura Zlati, Valentin Marian Antohi, Silvius Stanciu

**Affiliations:** 1 Department of Administrative Sciences and Regional Studies, Dunarea de Jos University of Galati, Galaţi, Romania; 2 Department of Accounting, Audit and Finance, Stefan Cel Mare University of Suceava, Suceava, Romania; 3 Department of Business Administration, Dunarea de Jos University of Galati, Galaţi, Romania; 4 Department of Finance, Accounting and Economic Theory, Transylvania University of Brasov, Braşov, Romania; 5 Department of General Sciences, Dunarea de Jos University of Galati, Galaţi, Romania

**Keywords:** Crude oil production, Crude oil import, Energy dependency, Energy balance, Energy crisis

## Abstract

The paper focuses on the impact of the complex global crisis on the European oil industry. The main objective of the research is to define, implement and validate a model able to quantify the developments and risks faced by this industry at European level. In order to achieve this objective, dynamic statistical analysis takes into account specific indicators of production, demand and actual consumption over a significant period of time. Special attention is given to the impact of the pandemic on this industry. The analysis takes into consideration the latest official statistical data and is connected to the most important global trends in the oil industry. The main result of this scientific approach is the building of a pertinent instrument/model able to assist the decision-makers in calibrating the European oil industry to global market requirements and developments. By using this tool, key elements of energy policy can be identified that can bring valuable clarifications in the context of the industry's new orientations towards green energy and the reduction of polluting fuels.

## Introduction

**Fig. 1:**
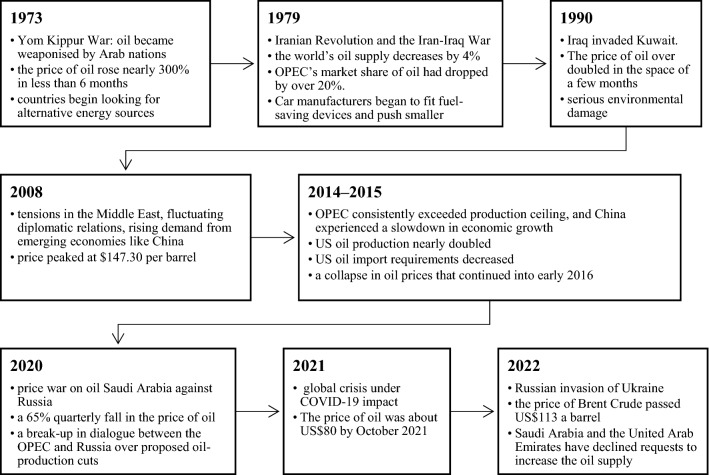
Historical background of oil crisis

Globally, and particularly in the EU, there have been numerous energy crises over the years. A summary of the most important of these challenges is presented in Fig. 1.

In the current geo-political context, like most major global economic players, the EU has had to adopt measures to reduce its dependence on oil imports, including from Russia. As a result, the EU has turned to the Green Deal strategy which also covers the deployment and development of renewable and unconventional energy sources.

At the end of 2021, against the backdrop of the global energy crisis, the prices of energy products including oil have risen dramatically, attracting galloping inflation not only in Europe. Amid Russia's invasion of Ukraine, Brent oil prices exploded, with the price per barrel surpassing $130 for the first time since 2008 (Disavino 2022).

Member States' reactions to these developments have been mixed. Thus, Belgium faced a price increase in the energy sector of 30–50% (Corroenne 2022).

France tried to implement anti-inflationary public policy and social protection measures that mainly targeted working families and immigrants. The cost of these policies has reached 580 million euros per year (Nussbaum and de Beaupuy 2021).

In the wake of the invasion of Ukraine, Germany has been trying to reduce oil imports from Russia, with a negative impact on oil-consuming industries. Moreover, other industries have also faced difficulties due to the embargo on Russian exports (Connolly 2022).

Greece has suffered enormously from the energy crisis. The effects have been felt particularly in the tourism sector. This is because the rise in price of crude oil, which supported a Unleaded Price increase over 2€/l (Kyriaki 2022).

In Spain, the price of electricity has risen by 200%, which has deeply affected the business sector and households, amid the halt in imports of energy sources from Algeria (Euronews 2021).

Romania has one of the safest positions in relation to oil imports: one third of what it needs is produced locally. At EU level, dependency is 96% (Petrescu 2022).

Energy independence represents in the current geo-political context a strategic element that can ensure economic independence in the context of an eventual geopolitical crisis. To achieve this goal, the EU has promoted an energy policy focused on sustainable components such as renewable energy consumption and the saving of environmentally polluting energy resources.

The dynamics of EU enlargement until the years 2000–2004 were based on the EU's monopolisation of countries with energy potential through European enlargement (Eurostat, 2021d). Since 2004–2005, the change of vision at European level has led to a gradual reduction in production and the Brexit phenomenon has led to a substantial reduction in European crude oil production capacity.

The geo-political context favoured Russia, as a country benefiting from rich energy resources, which had an upward curve of exports until 2005, after which there were consecutive decreases in the demand for energy products, leading the linear trend equation of Russia's exports to European countries in the period 2000–2019 to be characterized by the formula *y* = −0.0758*x* + 163.2 (slightly decreasing trend).

The same downward trend has been experienced by Libya as a significant energy supplier, but the trend is strongly downward (*y* = −2.6399*x* + 133.78).

A contrary development is shown by imports from Iraq (*y* = 1.4639*x* + 9.0907) and Nigeria (*y* = 1.0594*x* + 15.22), which offset the impact of lower Russian exports.

In structure, EU net imports underwent significant changes as follows (Eurostat 2021a):

- in the case of refinery feedstocks, net imports are significantly reduced from 23.6 million tonnes in 1990 to 8 million tonnes in 2020 (*y* = −1.8212*x* + 19.534);
- in the case of fuel oil, the net import balance decreased steadily until 2015, when the EU recorded a negative import balance (−14.2 million tonnes) as a result of European policies to reduce polluting energy consumption, but the trend could not be maintained. The EU is on an upward trend until the end of 2020, with a net negative balance of −5 million tonnes (*y* = −3.4261*x* + 14.688);
- the most spectacular reduction in consumption has been seen in the case of gasoline, in which case the negative import balance has been maintained on a constant downward trend (*y* = −8.6918*x* + 5.5286), from −6.3 million tonnes in the 1990s to −52.6 million tonnes in 2020, which contributes significantly (82%) to maintaining the EU energy balance at only 13 million tonnes in favour of net exports;
- positive trends in favour of imports were recorded for liquefied petroleum gases (*y* = 1.6512*x* + 2.3647); kerosene-type jet fuel (*y* = 2.6246*x*—5.7685) and gas oil and diesel oil (*y* = 2.1573*x* + 9.0304). Of all these imports, the most sinuous trend curve was recorded for the group gas oil and diesel oil, which serves the automotive industry and transport, objectives towards which the EU energy policy is mainly focused on the use of green energy and limiting the consumption of traditional fuels, which was successful in the period 205–2015, but which, with the onset of the pandemic, has experienced a revival amid the blockades of imports from China of electronic components needed for electric or hybrid cars, which has repositioned the European transport sector in favour of conventional means of transport.

In this context, it is noted that the economic crisis has had a significant impact on the European energy market, leading to increase in the prices of energy products and active measures to socially compensate for these increases, as well as technological transfer to conventional means to the detriment of new economic branches based on green energy consumption (green transport, green cars, green technologies, etc.).

It is therefore necessary to identify economic balances within the European market in terms of energy policy, sustainable economic growth and demand sensitivity to uncertainty. In this regard, we propose to develop a model able to quantify the developments and risks faced by this industry at European level, based on the use of industry-specific indicators and in the context of the challenges specific to the pandemic crisis.

The *objectives* of this research are:

- O1: Identification of the geo-political and economic context for a significant period of 30 years influencing the European energy market.
- O2. Identification of the main economic and energy development models debated in the literature.
- O3. Selection of representative statistical indicators, consolidation of a database over a significant period of 30 years.
- O4. Dynamic piloting of annual patterns and obtaining correlations of indicators in 30-year dynamics.
- O5. Analysis of correlations and dissemination of results with proposal of viable solutions.

The results of the research allow the identification and quantification of specific influencing factors on the European energy market and the provision of viable decision options for policymakers.

The novelty of the research lies in consolidating the dynamic picture over a significant period of time in order to capture all energy policy inflections, make consistent observations to inform policy changes in European energy policy and identify the effects of the pandemic on European energy market dynamics.

## Literature review

In the case of emerging countries, the analysis of the long-term causal effect between GDP and crude oil consumption is significant. Andrei et al. (2016) are taking this approach in the case of Romania in its efforts to support sustainable economic development as a solution to mitigate the impact of crude oil on the economy. Environmental protection and sustainable development can be achieved through environmental taxation even in the absence of direct connection between living standard, environmental protection, and degree of green freedom. The main conclusion of this analysis is very pertinent: “The pertinent design of the environmental taxation system represents one of the determinant objectives for inland policymakers, taking into consideration that during the analysed period, the importance of environmental revenues has increased both as gross values and share of GDP.” This approach was later echoed by Hájek et al. (2019), who state that introducing the carbon tax charged in the area of energy industries is environmentally effective.

The authors (Wang et al. 2016) conduct an analysis of the crude oil import market in the context of OIDCs based on a sample covering 38 countries. The used research method combines econometrics and complex network. The analysed indicators are indexes of dynamic topological structure and long-term global recurrence plots. The used method of analysis allows to highlight the global evolution and the characteristics of the of the crude oil import dependency. Research findings cover the entire crude oil import dependency system, masurarea risk in crude oil supply, highlighting the different role of countries in the crude oil import market and the fact that the crude oil import dependency system can be divided into different periods. European countries such as the Netherlands, Italy, Spain, Belgium, Austria, Poland and Germany had the highest average increases in their share of the crude oil market.

A global Oil Supply Chain (OSC) assessment model covers the period 2003–2013 and is carried out by Sun et al. (2017). The analysis covers China's crude oil imports and is based on a matrix that quantifies the availability, accessibility, affordability and acceptability risk. In the case of internal to external risk of oil imports, 2-dimensional matrix was used. As solutions for improving China's crude oil import policy, approaches capable of mitigating risk by decreasing the distance to import sources and sharing imports by source are listed.

Sharma et al. (2021) perform a crude oil demand analysis model in Southern Asia using Pooled Mean Group (PMG) estimation. Analyses quantifies the impact of crude oil price, income, primary energy sources, electricity and financial crisis on the demand for crude oil six countries. The analysis covers the period 1988–2016 and leads to the conclusion that there is a positive influence from GDP growth and primary energy sources on demand for the crude oil.

The impact of the change in the crude oil trade pattern on economic development was realised by Xi et al. (2019). Analiza acopera 65 countries along the Belt and Road si aduce ca element de noutate the relationship between trade and GDP from the perspective of trade relations. A first conclusion of the research is related to the different position of the states within the B&R and the clusters formed. A second conclusion concerns the positive effect that the changes in the crude oil trade pattern have had on the economies of B&R countries. In fine, the impact of changes in the crude oil trade pattern on their economies is different. In this context, Romania is mentioned for its role as a hub of the Belt and Road to enhance its value.

The mathematical approach to crude oil distribution can be optimized by minimizing the total trade cost in the conception of Dong et al. (2020). The authors propose a complex network theory able to offer solutions on exporting and importing countries. Within this network, the oil trading countries are nodes capable to satisfy their own supply and demand structure.

An interesting research (Reuter et al. 2019) considers the impact of policies that may alter final energy consumption for the European Union and its member states. In particular, the analysis focuses on the cases of Germany and Poland over the period 2000 and 2015 based on a logarithmic approach. The industries analysed in terms of final consumption are as follows: residential end-uses, transport modes and industrial sub-sectors. The main conclusion of the analysis highlights that final energy consumption in the EU28 is strongly influenced by an increase in energy efficiency in industry followed by households. Germany is facing to relatively low energy efficiency improvements in industry but strong energy efficiency gains in households followed by transport, while Poland is facing to a strong increase in final energy consumption. A similar approach (Tsemekidi Tzeiranaki et al. 2019) analyses final energy consumption in residential sector and notes that consumption peaks were reached in 2010, 2014 and 2016. The authors consider that technological and policy changes over the period of 2000–2015 at EU level have resulted in” a constant annual sFECc, decline rate of 0.0127 ktoe/m^2^/thousand EUR”. Addressing energy efficiency in industry is done by Malinauskaite et al. (2019). According to the authors, the best energy efficiency in European industries, amid energy policy changes, are recorded by Germany, France, Italy and Spain.

Tovar-García and Carrasco (2019) theorize that export and import composition affects incomes and price elasticities and trade balance. In the case of crude oil, import price inelasticity is dependent on Russian trade balance in goods. As a result, the increase in Russian imports of high-tech products influences the level and price of Russian crude oil exports to Europe.

Crude oil remains an exceptionally important resource for the EU economy, which is heavily dependent on imports. Horobet et al. (2019) study in this context the dependence between oil and stock prices, in the context of using macro-indicators such as local stock market indices, the EUR/USD exchange rate, the oil imports dependency, inflation rate, and global volatility indices. The results of the analysis show that the EU is exposed to oil price changes over the long run and to global energy market shocks. On the other hand, the authors state that there is a different behavior of market investors over the short versus the long run concerning the valuation of financial companies’ stock prices in relation to oil price.

The optimization of crude oil-supply portfolio and energy security is analysed by Sun et al. (2020). The analysis is based on an optimization model that follows minimizing cost and risk, using a decomposition hybrid interval prediction model. The model has been applied to forecasting the volume of China's crude oil-supply. This research is an effective tool for decision-makers because it quantifies several types of risk and produces several scenarios of evolution. The model can be used for enterprises investment decision-making and government planning. The forecast model shows that the total cost of imported crude oil fluctuates sharply while the total risk of imported crude oil goes relatively stable. The authors propose that decision-makers pay more attention to countries with greater cost and risk flexibility: Iran, Iraq, and Qatar.

Establishing crude oil price regulations and quantifying their impact on imports are discussed by Chen and Sun (2021) with reference to China. The approach is aimed at how the Chinese economy responds to price fluctuations in the global crude oil market. A first conclusion of the study shows that gasoline prices in China show an asymmetric response to the international crude oil price. This response takes the form of a strong, rapid response to crude oil price increases. Another conclusion is that state-owned refiners responding more to a regulated price increase. Finally, in the context of crude oil import regulation, China’s gasoline price responds symmetrically to fuel oil price changes but asymmetrically to price regulation.

A forecast to the year 2060 of global energy consumption according to the scenarios proposed by the World Energy Council was made by Kober et al. (2020), which shows that the energy future is destined for a moderately increasing consumption, while the rate of final consumption will increase even more. Energy consumption is expected to double over the next 40 years. As far as oil and gas consumption is concerned, it is estimated that it may increase in the next period (horizon 2060) when the global population is estimated to reach 10 billion, due to energy security policies. The same approach is taken by Mandley et al. (2020), who advocate replacing crude oil consumption with bioenergy and other non-conventional sources in order to reduce the EU's energy dependence by 2050, improve cohesion in European energy policy, and thus have positive effects on trade and energy security.

Hosseinabad and Moraga (2020) start from the idea that energy security is one of the greatest challenges of the current period for developed countries in their efforts to reduce energy dependency. The authors make a connection between electricity/energy consumption, renewable energy resources as promotion plans, and energy dependency on imported resources, based on 3 scenarios applied to the state of Illinois in the USA. The central objective of the analysis is to find real possibilities to reduce energy dependency and implicitly crude oil imports. The 2025 forecast analysis concludes that reducing imports of primary energy sources can generate savings of $17 billion.

In the case of the USA, an analysis on decreasing energy dependence by Mourdghaffari et al. (2020), who starts from the idea that energy policy should focus on domestic energy sources, including crude oil, while modernizing energy systems and energy efficiency. This is in view of the fact that North America has the largest share of crude oil production and supply in the field of unconventional resources. The introduction of new technologies has led to increased oil and gas supply and production power.

The EU's dependence on crude oil has been analysed by McGovern et al. (2018) over a 20-year period. The analysis concludes that the EU had a dependence on crude oil imports of 96% of supply in 2018. This was due to the fall in crude oil production in the Member States to a much greater extent than the fall in consumption. Dependence on crude oil has also increased as a result of Brexit, with the UK accounting for 70% of EU crude oil production in 2018. Denmark, Italy, Romania, Germany and Poland are the only Member States with output of crude oil greater than 1 Mt per year. With the exception of Denmark, Romania, Hungary and Croatia, Member States cover 90% of domestic crude oil supply from imports. These imports come from Russia (31% of total crude oil import volumes), Middle Eastern and African OPEC members. In order to mitigate the risks associated with crude oil imports, the Oil Stocks Directive was issued, obliging Member States to hold stocks of oil to reduce the effects of supply shortages.

Ensuring the continuity of crude oil supply in Poland is analysed in a research conducted over the period 2000–2018 by Kamyk et al. (2021). This approach shows that primary energy sources are decreasing over the period analysed, while consumption is increasing, especially for natural gas and other primary energy sources. As a result, Poland's imports of crude oil are steadily increasing, currently covering 97% of the population's consumption. Poland's energy policy aims, on the one hand, to diversify the supply of crude oil and, on the other hand, to reduce imports from risk areas. In the medium term, the authors make a forecast showing that alternative energy sources (biofuels, alternative fuels and electro-mobility) will not develop at the same pace as in Western Europe, which will cause the demand for crude oil in Poland to grow further.

A broad analysis of the evolution of crude oil supply and demand in the USA by Monge and Gil-Alana (2021) covers the period 2007–2020, when the oil market covers 8% of GDP. The authors develop a model based on fractional integration, fractional cointegration VAR and wavelet analysis. In the long run, the analysis concludes that there is a balance between the data series analysed. Furthermore, the implementation of the model allows the delimitation of geographical areas affected by an increase in the shale oil production, which is followed by a decrease in WTI crude oil prices.

The EU's efforts to reduce energy dependence on imports have also been reflected in the European Green Deal 2050 project. From the perspective of Leonard et al. (2021), this project will have a positive impact on the EU energy balance by reducing crude oil imports and promoting sustainable energy sources. Moreover, the authors mention the efforts made by the European institutions to achieve a global cooperation in the field of energy that is able to bring benefits to all countries of the world. The Green Deal approach from the perspective of the diverging interests of the Member States is carried out by Hafner and Raimondi (2020). The authors introduce unconventional elements into the analysis, such as European citizens' fear of losing their jobs in traditional energy industries, Russia’s repositioning of crude oil exports to the EU, and increased risk in areas from which the EU imports crude oil.

The economic situation, energy imports, and energy demand in the EU are analysed by Rokicki and Perkowska (2021), who find that exports and production are medium and weakly correlated. The authors believe that the economic factor has less impact than energy policy, especially as EU energy imports are less volatile than its exports. As a result, energy security is an important target at EU level.

The impact of oil prices and rapid urbanisation on the environment is strong in the opinion of Mahmood et al. (2022). The authors propose and implement a nonlinear autoregressive model that considers Gulf Cooperation Council (GCC) countries over the period 1980–2019. The findings of the study are interesting. Among other things, the authors note that the rising oil price has a positive impact on CO_2_ emissions. These emissions also depend on the level of economic development in the countries analysed.

In the context of the challenge of sustainable development (Yu et al. 2022a, b) analyse energy consumption in Organization for Economic Cooperation and Development countries. The authors apply nonlinear methods and point out the Jevons' paradox in these countries. The main conclusion of the analysis is that the technological innovation becomes a factor that mitigates energy demand.

An interesting approach is the one which analyses the correlation between crude oil imports, renewable energy, transport services, trade, industrial value-added, and patents (Yu et al. 2022a, b). The analysis covers the German economy from 1990 to 2020. The authors are using an Autoregressive Distributed Lag model. The authors conclude that there is a strong correlation between the economic indicators analysed and crude oil imports of Germany. In the case of renewable energy, the connection with crude oil imports is reversed.

The use of business models in the context of Industry 4.0 technologies and the Circular Economy (CE) covers 213 automotive firms in Eastern Europe by (Khan, Umar, et al. 2022). The authors believe that these models can suggest the importance of the circular economy in sustainable development and thus in reducing crude oil consumption. Circular economy in the context of decreasing consumption of solid fuels is the subject of other research (Khan, Shah, et al. 2022b).

The use of second-generation econometric techniques by Khan et al. (2022a, b, c) is in the context of analysing economic growth by groups of states by income level. The authors consider that the main source of economic development is the intensive use of factors. Moreover, current economic development must be sustainable and involve a reduction in crude oil consumption, especially in the post-COVID-19 period.

The Circular Economy (C.E.) practices and the improvement of product delivery services under COVID-19 impact in food sector supply chain from Ecuador are analysed by Khan et al. (2021). The authors believe that resilience in uncertainty and risk scenarios can enable sustainable development in which fossil fuel consumption is drastically reduced.

An interesting analysis by Rehman Khan et al. (2022) covers nine top exporting countries. The analysis takes into account specific indicators such as energy efficiency, renewable energy consumption, foreign direct investment, logistics industry, manufacturing industry and global trade during the COVID-19 pandemic. The authors focus on the supply chain operations globally.

From the study of the literature, it appears that there is a major concern of Member States and other countries of the world to reduce pollution and transfer to clean renewable energy consumption. In this context, energy dependence remains a topic of major interest, especially in the context of projected increases in final energy consumption across all four components (industrial, commercial, services and household).

Even if in the literature there are models for assessing and forecasting energy consumption, we have not found a current model that scales crude oil production in relation to energy dependence. We believe that our approach is timely and justified in relation to the Energy Security and Pollution Reduction objectives assumed by global players.

## Methodology

From the methodological point of view, the research covers all stages of an experimental work, starting with data collection from international databases over a period of 30 years (Eurostat 2021a, b, c, c, f) consolidation of the databases, transformation of the indicators to ensure comparability, analysis of the dynamics of the indicators through descriptive analysis procedures, comparison of the correlation of the data series in dynamics through regression analysis and graphical analysis (elaboration of partial correlation diagrams), according to the Annex.

Domestic crude oil production is a significant indicator of conventional energy market dynamics. This indicator quantifies on the one hand the size of national resources, the quality of their exploitation and the adherence to energy policies that currently focus on the less polluting green energy component. In this regard, we have established this indicator in which the dynamic component has been standardized to the level of the overall average for easy to interpret and present modelling synthesis, namely Primary production of Crude oil (Thousand tonnes of oil equivalent), calculated by relating the unit value at country and year level to the EU annual average of the respective year—PRIMPROD, as the dependent variable of a generalized econometric model of energy market correlation analysis.

We selected three other impact components to quantify:

- energy dependency, which identifies the regional distribution in terms of energy policy and the orientation of the European economy towards sustainable sectors through dynamic standardisation at the level of the overall average evolution for the 30 years analysed, i.e. EIP (Energy Imports Dependency, calculated by relating the unit value at country level to the EU average);
- the level of imports, which identifies the gap between demand and domestic production of crude oil through dynamic standardisation at the level of the general average evolution for the 30 years analysed, i.e. SIEC (Imports of oil and petroleum products by EU countries according to Standard international energy product classification, calculated by relating the unit value at country level to the EU average);
- Gross Domestic Product—GDP evolution at national level on average of the evolution trends in the period 1990–2020.

From the literature, some significant issues have been identified regarding the energy dependence of Member States in relation to crude oil production and imports:

Hájek et al. (2019) appreciate that the taxation of pollution in the European energy industry has a direct effect on sustainable development. According to (Malinauskaite et al. 2019; Reuter et al. 2019; Tsemekidi Tzeiranaki et al. 2019), final energy consumption in the EU is strongly influenced by increasing energy efficiency in industry and households. Over the next 40 years, global energy consumption will grow moderately as final consumption grows faster due to the introduction of unconventional energy sources (Hafner and Raimondi, 2020; Kober et al. 2020; Leonard et al. 2021; Mandley et al. 2020). In this context, we define working *hypothesis H1: The energy dependence of Member States on oil production is in a lower correlation the more technological progress and the use of green technologies are developed*.

According to Kamyk et al. (2021), the development of alternative energy sources will create disparities between Western and Eastern EU in the medium term. According to Sun et al. (2017), improving crude oil import policy is achieved by decreasing the distance to import sources and sharing imports by source. Approaching crude oil imports through network theory offers solutions to crude oil exporting and importing countries in the view of Dong et al. (2020). 31% of European crude oil imports come from Middle East Russia and African OPEC countries. Dependence on crude oil has increased as a result of Brexit (McGovern et al. 2018). Rokicki and Perkowska (2021) believe that at EU level, crude oil exports and production are moderately or weakly correlated. Based on the above, we define working *hypothesis H2: The energy dependence of Member States on oil imports from within the EU is all the more reduced as policies to limit consumption and exploitation of traditional fuels are more strongly promoted in favour of green energy.*

Sharma et al. (2021) conclude that GDP growth has a positive influence on demand and crude oil prices. Wang et al. (2016) suggest the dynamics of European countries' position on the global crude oil import market. Xi et al. (2019) support the same approach, but with reference to B&R countries. Andrei et al. (2016) appreciate that sustainable economic development reduces the impact of crude oil production and imports on the economy. The EU is exposed to long-term oil price changes and global market risks as stock market investors shift their focus to global equity prices according to (Horobet et al. 2019). This aspect should be considered in relation to the inelasticity of the energy consumption function. This approach allows to define the working *hypothesis H3: Energy dependence is an indicator weakly influenced by the correlation with the national GDP deflator, tending towards 0 in conditions of economic crisis.*

We define the following model to quantify energy dependence risk through indicators of crude oil production, imports and economic growth:

1 $$ {\text{PRIMPROD}}_{i,t} = \alpha_{i,t} *{\text{SIEC}}_{i,t} + \beta_{i,t} *{\text{GDP}}_{i,t} + \gamma_{i,t} *{\text{EIP}}_{i,t} + \varepsilon_{i.t} $$

where PRIMPROD—Primary production of Crude oil (1000 tonnes of oil equivalent), calculated by relating the unit value at country and year level to the EU annual average for that year; EIP—Energy imports dependency, calculated by relating the unit value at country and year level to the EU annual average for that year; SIEC—Imports of oil and petroleum products by EU countries according to Standard international energy product classification, calculated by relating the unit value at country and year level to the EU annual average for the year in question; GDP—Gross Domestic Product (current EUR)—calculated by relating the unit value at country and year level to the EU annual average of the respective year.

*α,β,γ*—regression coefficients; *i*—number of states for which the risk of energy dependency is determined; *t*—the number of years for which the analysis is performed.

At the European level (EU27), the regression function was determined for *t* = 30 years, i.e. the period 1990–2020, taking into account the averages of the evolution of the indicators (standardization of the trend dynamics) obtaining the following regression equation using the least squares method and the multiple linear regression function:

2 $$^ \wedge{\text{PRIMPROD}} = 0.520* {\text{SIEC}} + 0.282*{\text{GDP}} - 0.396* {\text{EIP}} + 22.454 $$

The statistical analysis of the consolidated database after standardization of the trend dynamics reflects for the proposed general model a statistical significance of about 80% of the model, which means that the risk of energy dependency is determined in the model equation in 80% of the analysed cases.

The standard error of the regression point indicates a value of 0.98, which allows to validate the model together with the results of the change statistics, namely the correlation coefficient Sig of the function *F* = 0, and the Durbin-Watson coefficient tends to 2. The statistical tests are shown in below Table 1.

**Table 1:** Model summary

Model	*R*	*R* square	Adjusted *R* square	Std. Error of the estimate	Change statistics	
					*R* square change	*F* Change
General	0.892^a^	0.795	0.769	0.98805	0.795	29.797

Model	Change statistics			
	df1	df2	Sig. *F* Change	Durbin-Watson
General	3	23	0.000	1.989

Dependent Variable: PRIMPROD

^a^Predictors: (Constant), GDP, EIP, SIEC

By ANOVA test, the rejection of the null hypothesis was verified by calculating the one-sided critical probability, which result is favourable to the rejection of the null hypothesis (Sig coefficient level being lower than the chosen significance threshold of 0.05), with the calculated *F* function value of 29.797 points and a quadratic mean of 29.089 points. From the sum of squares test, the assimilated value of the regression function is strictly larger than the residual component, which allows a positive assessment of the homogeneity of the data, the number of degrees of freedom of the regression point being equal to the number of regression variables proposed for the model, no variable being excluded. These aspects are presented in Table 2.

**Table 2:** ANOVA test

Model	Sum of squares	*df*	Mean square	*F*	Sig
General					
Regression	87.268	3	29.089	29.797	0.000^a^
Residual	22.454	23	0.976		
Total	109.722	26			

Dependent Variable: PRIMPROD

^a^Predictors: (Constant), GDP, EIP, SIEC

**Fig. 2:**
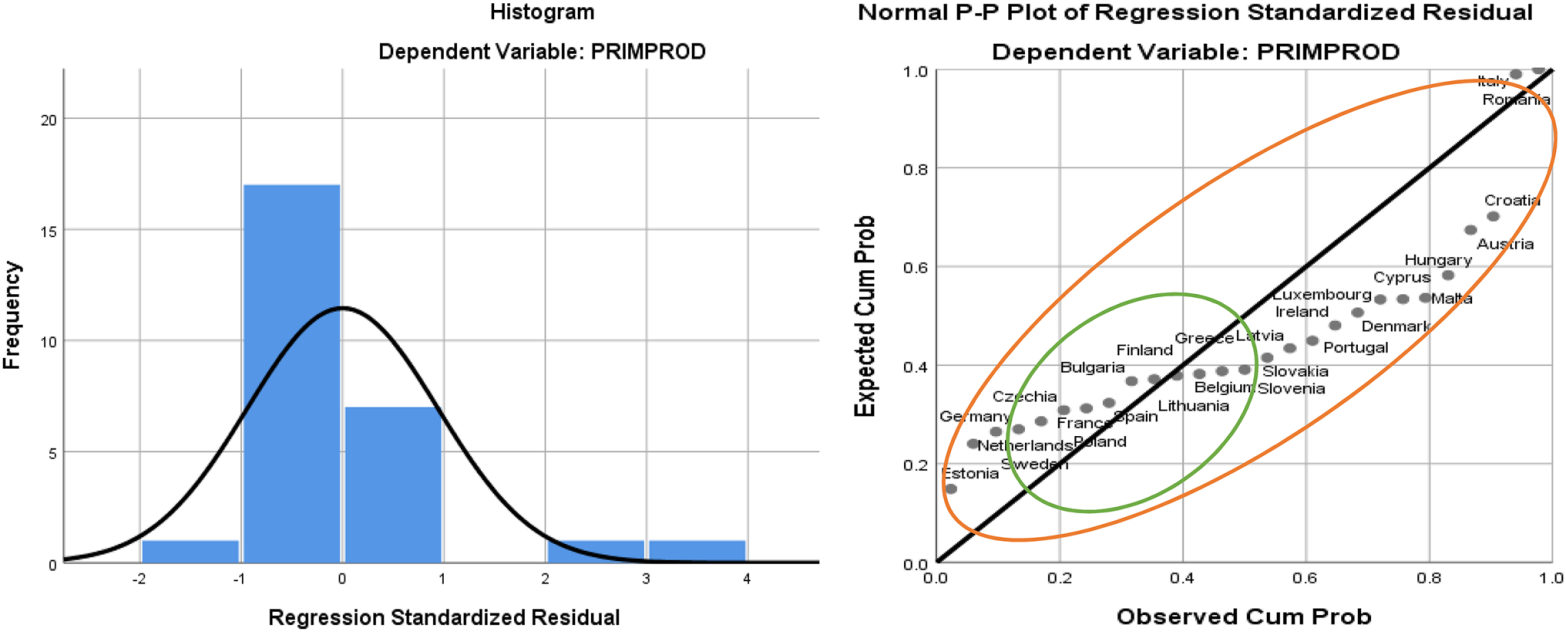
Graphical method for analysing the risk of energy dependency in the EU27

Using the graphical method, it can be seen that the histogram distribution generally falls below the Gaussian curve, with a small deviation in the upper part of the ascending slope, and the projection of the P–P Plot reflects the territoriality of the risk of energy dependency, with different deviations from the trend line for developed member countries such as France, Spain, Belgium, Finland compared to less developed member countries (Cyprus, Hungary, Croatia, Latvia, Estonia, Romania), (see Fig. 2).

**Fig. 3:**
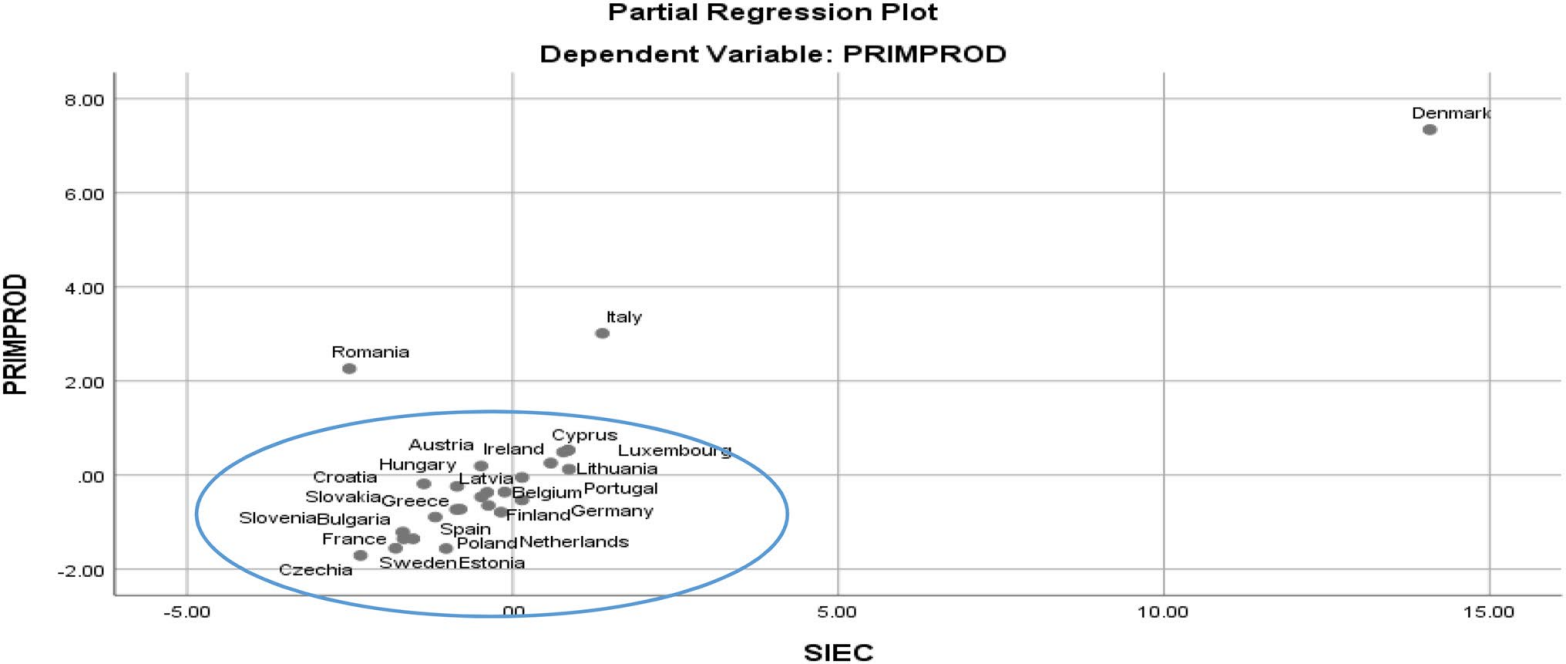
Partial dependency risk correlation diagram for the crude oil production–consumption function

Partial correlations of the dependent variables (crude oil production) with the model regressors assess the territorial risk on each component. As regards the correlation between production and imports, as a factor for estimating risk, there is a cluster agglomeration that highlights the unitary behaviour assimilated to the production–consumption function, although there are some exceptions for countries such as Romania, Italy and Denmark. We assess that these countries are exposed to a lower risk of energy dependence (Fig. 3).

**Fig. 4:**
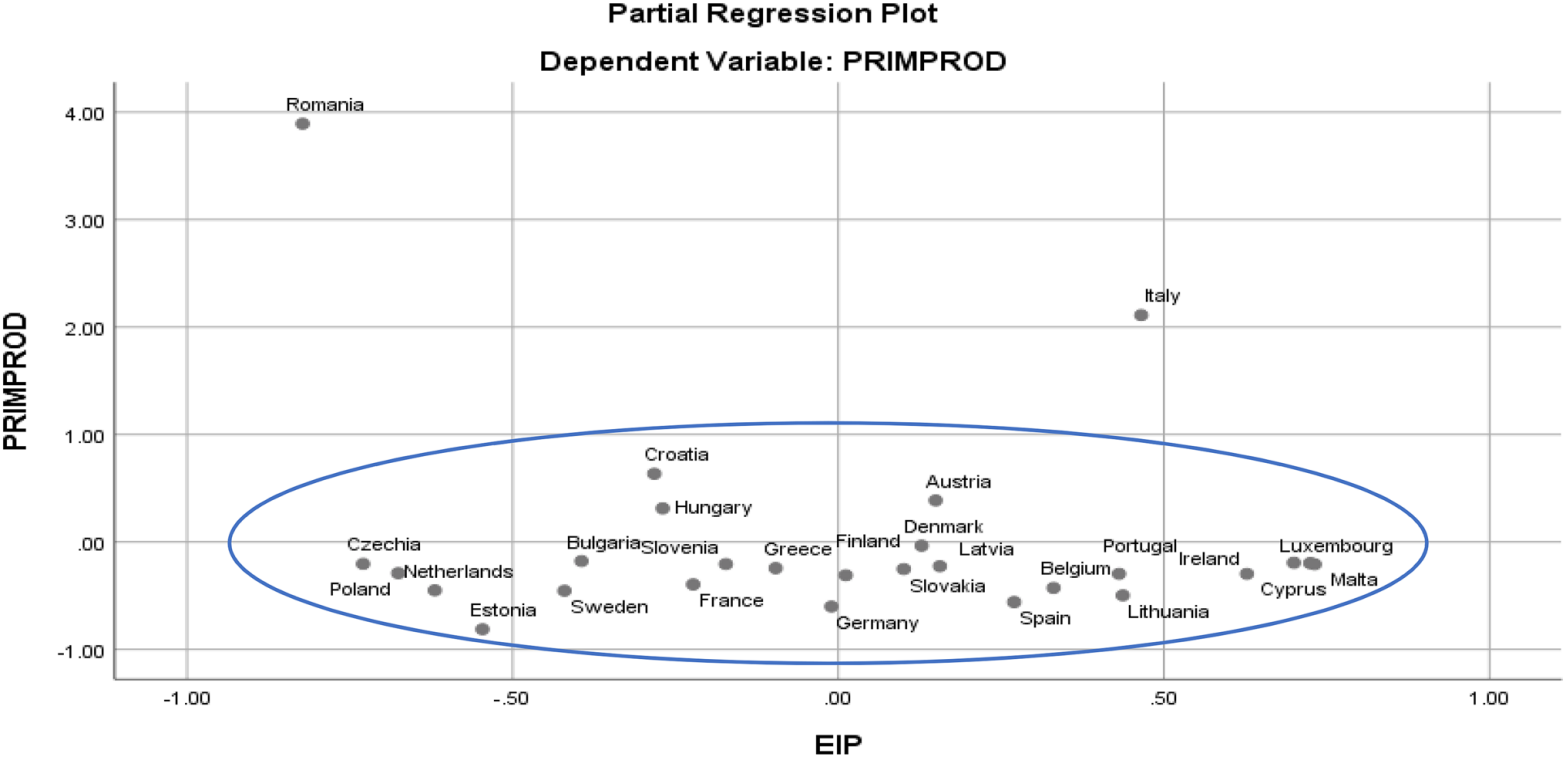
Diagrama partial de corelatie a riscului de dependenta pentru functia productie de crude oil-dependenta energetica

From the production-energy dependence correlation point of view, it can be seen from Fig. 4 that Denmark re-enters the cluster, leaving only Romania and Italy outside, which leads to the idea that the risk of energy dependence even for countries exposed to it is differentiable by levels of intensity.

**Fig. 5:**
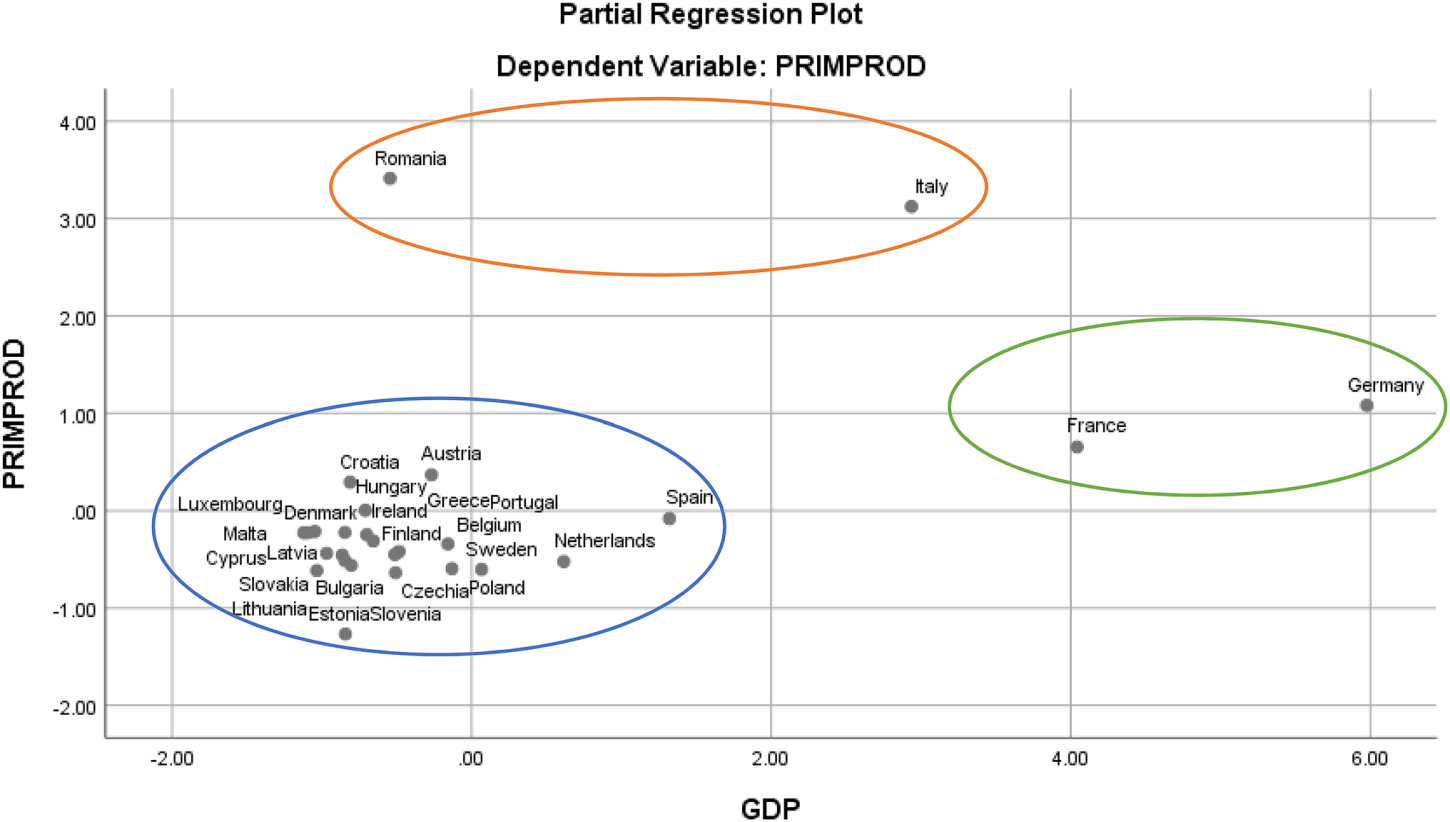
Partial dependency risk correlation diagram for crude oil production function-GDP, economic growth

With regard to the risk of energy dependence in terms of economic growth and crude oil production, it can be seen that the poles of economic growth in the EU27 (Germany and France) tend to form a distinct cluster, which is moving out of the energy dependence risk zone, while Romania and Italy remain outside the general cluster, in the minimum energy dependence risk zone (see Fig. 5).

The proposed model identifies the risk of energy dependence as a component based on the reduction of crude oil production directly correlated with the decrease in imports, so if a unit of production covered 0.5 units of imports, it is found, based on the trend of evolution of the indicators, that the degree of dependence is doubly influenced, reflected by the negative value of the EIP coefficient of the econometric function. In the current conditions of green energy promotion, the risk of dependence remains high, and causal analyses are needed to highlight solutions to reduce this risk.

## Discussions

The current geopolitical context and the succession of energy crises presented in the Introduction section show that energy balance has become more of a desideratum than a reality. In addition, the impact of imbalances is likely to destabilise national economies, given that oil consumption currently underpins multiple production processes and the support of logistics chains, most of which are car-based. The TEN-T corridors, a European desideratum, are not fully functional due to infrastructure problems in Eastern European countries. This too has repercussions on functional logistics chains.

Within these parameters, the European Commission's efforts to gradually reduce oil dependency are proving elusive, even though substantial funds have been allocated for this purpose. We appreciate that the proposed model for quantifying the risk of energy dependence is a very useful tool for European states and not only for identifying viable solutions in line with the Green Deal objectives.

**Fig. 6:**
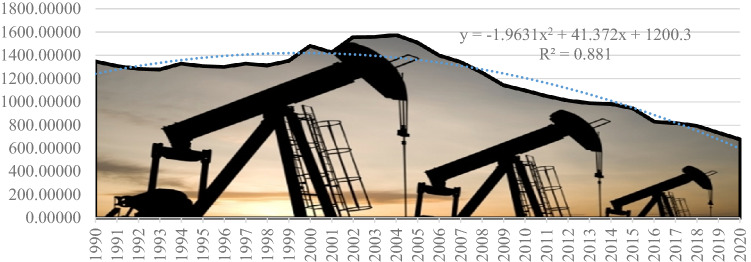
European primary production trend (thousand tonnes—crude oil) developed by the authors using (Eurostat 2021c)

The analysis showed that the European average crude oil production in the period 1990–2020 was on a downward trend characterized by the second degree polynomial equation: *y* = −1.9631*x*^2^ + 41.372*x* + 1200.3. The statistical significance level of the trend function is 88%, which confirms that the downward trend is in line with current policies to curb pollution and transition to the green economy. Until sufficient green energy supply stabilises, we estimate that this trend will contribute to the risk of energy dependency at EU27 level (Fig. 6).

**Fig. 7:**
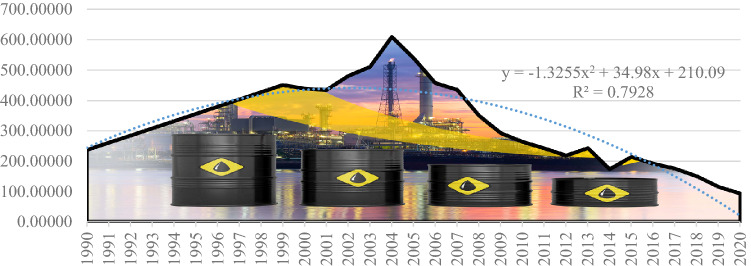
Imports of oil and petroleum products trend developed by the authors using (Eurostat 2021e)

As far as crude oil imports are concerned, they have on average recorded a downward trend, the European policy being to compensate the reduction of oil production by imports up to the level of 2005. After this date, imports have been steadily reduced, as European economies are now in transition to the green economy, which induces the second component of dependent risk, characterized by the downward trend line of imports according to the polynomial equation *y* = −1.3255*x*^2^ + 34.98*x* + 210.09. The coefficient of determination is lower than in the case of the import trend line (79.28%), due to energy policy changes adopted by Member States up to 2005 (see Fig. 7).

**Fig. 8:**
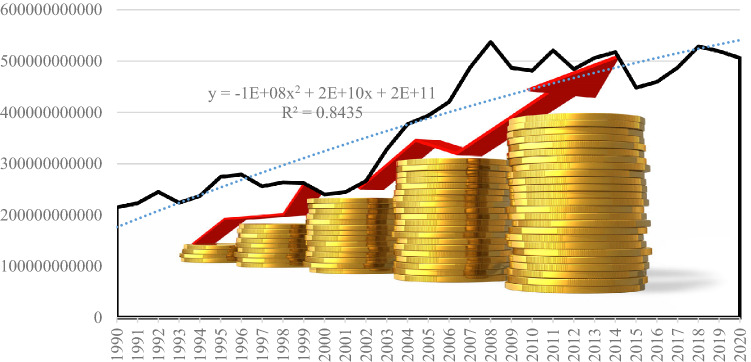
GDP trend (EUR) in the European Union developed by the authors using (Eurostat 2021f)

The analysis of economic growth in terms of its contribution to ending energy dependence reflects the fact that at EU27 level the upward trend has been disrupted by multiple crisis situations. The most significant are the economic and financial crisis triggered in 2008 by the bankruptcy of Lehman Brothers Holdings Inc and the current crisis triggered by the Covid 19 pandemic (see Fig. 8).

**Fig. 9:**
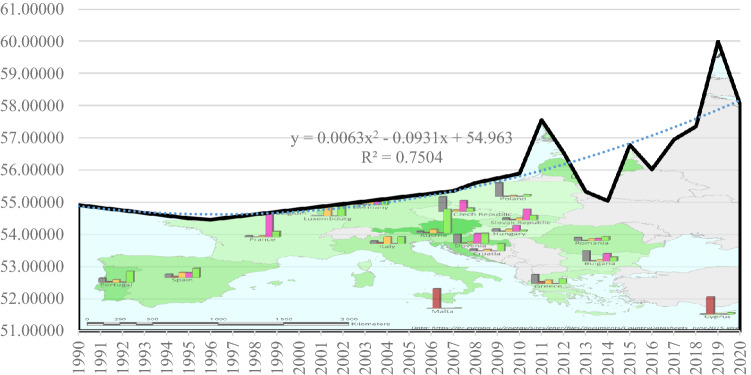
Energy dependence trend in the European Union (EU27) developed by the authors using (Eurostat 2021b)

Based on the above results, we can quantify the risk of energy dependency by means of a polynomial trend function with ascending character driven by energy policy changes and the transition to the green economy at EU27 level. The polynomial risk function is *y* = 0.0063*x*^2^—0.0931*x* + 54.963. It can be seen that the risk is increased in the period 2015–2020 both against the background of reduced primary energy consumption, not enough green energy, but also against the backdrop of the onset of the pandemic, which has favoured the transition to the digital economy, another area likely to increase the risk of energy dependency (Fig. 9).

**Fig. 10:**
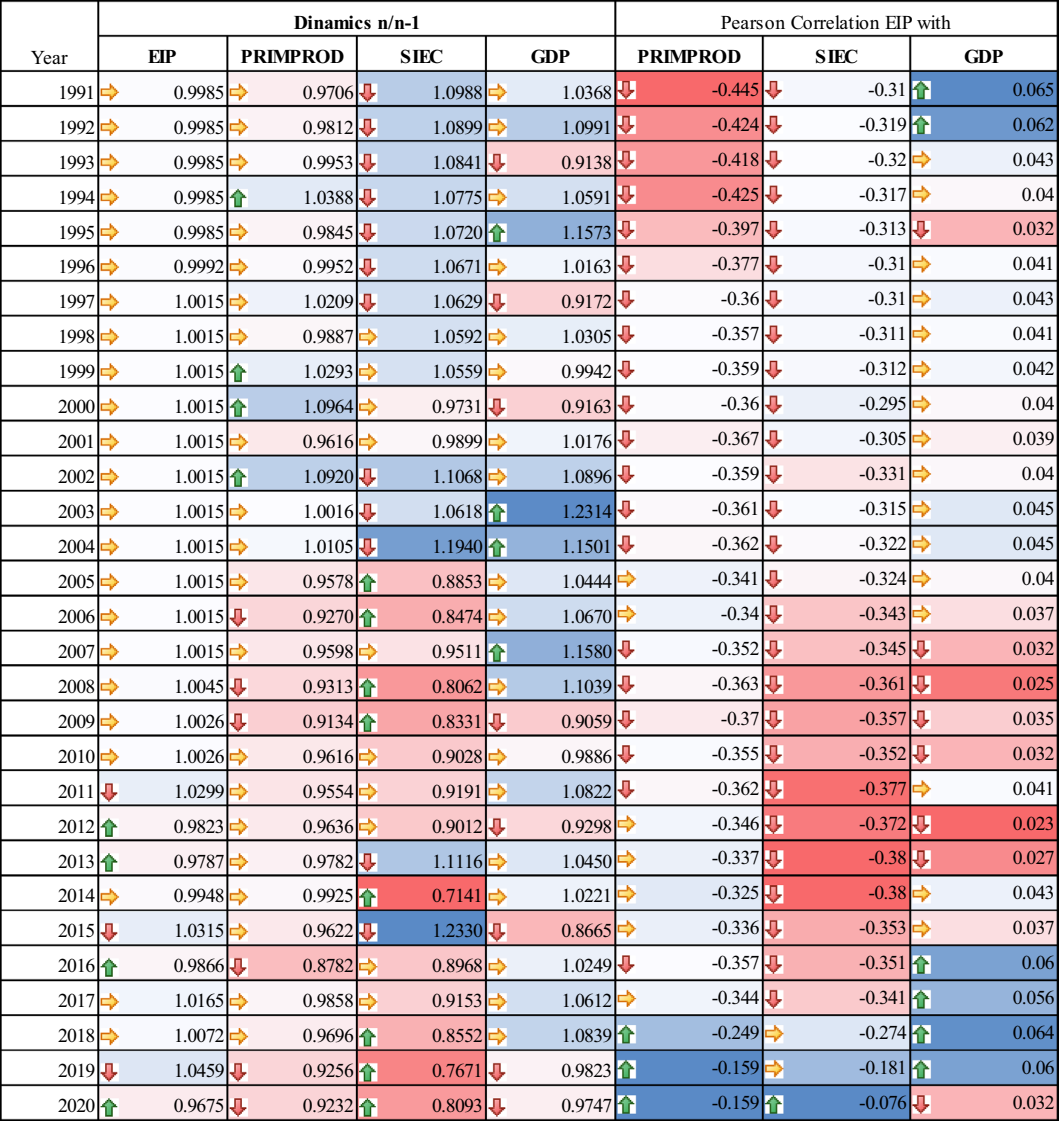
Histogram distribution of Pearson correlations and annual moving averages of energy dependency risk quantification model indicators

The development of the risk model proposed in the Methodology section for the non-standardised dynamic data series allowed the projection of Pearson correlations for the model variables in dynamics over the period 1990–2020 on average at EU27 level. The data are centralized in the histogram distribution in Fig. 10.

This approach allows validation of the working hypotheses as follows. In the period 1990–1995, the correlation between energy dependence and oil production followed the classical need-consumption curve in the sense that one unit of production decreased the level of dependence by up to 0.45 units. In the next 10 years, 1996–2005, there is a reduction of about 25% in the degree of dependence on imports, which is also favoured by an average increase in about 1.5% per year in domestic oil production, the use of new technologies in the exploitation of oil fields, EU enlargement, etc. Since 2006, until 2011, the period of pre- and economic crisis in the EU, the correlation of energy dependence on domestic production is decreasing, reaching the threshold of 0.34 units of reduced dependence per unit of production. This is mainly based on the approximately 5.8% annual reduction in oil production, a trend that continues to date, but the causalities are different. Between 2006 and 2011, the main causes of domestic oil depletion were financial gridlock, the economic crisis, private company bankruptcies and inflation. Over the period 2012–2020, the greatest shift in energy dependence away from domestic oil production is observed. This period is characterised by the promotion of green energies, increasing support measures for the development of green industries both upstream (automotive industry, digitisation, etc.) and downstream by promoting the green energy industry, exploiting renewable energy sources instead of the polluting oil industry. This approach validates H1: Member States' energy dependence on oil production is in a lower correlation the more technological progress and the use of green technologies are developed.

As far as imports are concerned, energy dependence has experienced a decelerating trend in correlation with this indicator. Thus, in the period 1990–1995, the level of energy dependence was reduced by 0.3 units per unit of imports, while the level of imports was overunity in terms of dynamic analysis, but with a decelerating trend, from about 10% growth rate in 1990 to about 7% growth in 1995. In the period 1996–2005, the level of correlation of energy dependence on imports is maintained with small variations within the limit of 0.3 units of dependence for one unit of imports. On the other hand, the dynamic import indicator also reaches sub-unit values, with an average overall import growth of 5% per year. In 2005, there was the largest reduction in imports compared to the previous year, 11.5%. In the period 2006–2011, energy dependence increases its correlation with the imports indicator against the background of decreasing domestic production, with the mention that the dynamic value of the indicator is subunitary, the average deceleration of imports being 12.5% in the period before the global economic crisis. Between 2012 and 2017, the production deficit continues to be counterbalanced by the increase in imports, with two significant peaks in the increase in the quantity of imported oil, 23% in 2015 compared to 2014 and 11% in 2013 compared to 2012. The correlation level of dependence on imports is on average, during the period analysed, a reduction of 0.36 points of dependence per unit of imported product. The average level of import dynamics is reduced by 4% annually, with large fluctuations in the import policy for petroleum products over the period. From 2018 to the present, energy dependence is increasingly weakly correlated with the import indicator, reaching the level of reduction of only 0.07 units of dependence per unit of imported product, and the level of imports is decreasing, with an average reduction of 19% per year. This approach validates H2: Member States' energy dependence on oil imports from within the EU is reduced as policies to limit consumption and exploitation of traditional fuels are more strongly promoted in favour of green energy.

A low level of correlation between energy dependency and GDP growth was observed between 1990 and 1995. Thus, although overall GDP growth during this period was 5% annually, energy dependence fluctuated between 0.06 units per unit of GDP growth in years with GDP growth up to 5% and 0.03 units of energy dependence per unit of GDP in 1995, when GDP growth was 15% at EU level. In the run-up to the economic crisis in 2008, there were no major changes, with an average dependency of 0.04 units per unit of GDP growth. From 2008 onwards, a reduction of the correlation level to 0.02 degrees of dependence on an additional unit of GDP is observed during the economic crisis and until 2013, thus the whole period is marked by GDP stagnation and economic growth. The average value was 1.0093 units of dynamic GDP growth. From 2013 to 2019, as the economic crisis calms down and the economy returns to normality, characterized by an average GDP growth of about 1.2%, there is a slight increase in the correlation of energy dependence with GDP to the 1990 value, i.e. 0.06 units of dependence per unit of GDP growth. In the years 2019–2020, against the background of the economic crisis, there is a worsening of both the evolution of GDP (economic recession characterized by a negative GDP rate of 2.15% annually) and the reduction of the degree of dependency is achieved at the level of 2007, i.e. 0.03 units of dependency for an increase in one unit of GDP. This approach validates H3: Energy dependence is an indicator weakly influenced by the correlation with the national GDP deflator, tending towards 0 in conditions of economic crisis.

## Conclusions

The main objective of the research was to define, implement and validate an energy dependency risk model based on the correlation with the main economic indicators of the sector at EU27 level.

The authors achieved the specific objectives of the research and identified the geo-political and economic context for a significant period of 30 years influencing the European energy market, i.e. an EU dominated by the sensitivity of energy dependence on imports from Russia and the Middle East, but on the other hand, concerned with reducing pollution and implementing new sustainable development strategies through directives. The main economic and energy development models debated in the literature have proven to be those focused on green technologies, and there are concerns among specialists about the degree of energy dependency and the minimum level of security from which current European energy policies start.

The next three objectives were to design an econometric model. The proposed model, based on statistical analysis over a period of 30 years, has generated significant and valid results, which indicate that in the context of the reduction of primary energy use, against the background of the EU27 transition to the green economy, the risk of energy dependence increases, and differentiated measures are needed at the level of each Member State to avoid a risk situation and an energy crisis with a strong impact on the whole economy.

The authors showed that the risk function is maximized in the current period defined by the pandemic crisis and the transition to the green economy, but the risk function is differentiated at the level of European countries, with a main cluster reflecting the position of the EU in relation to energy sector issues, a cluster with a higher level of Security against the background of accelerated economic development (represented by Germany and France) and a cluster less exposed to the risk of energy dependence, which includes Italy and Romania.

The relevance of the study lies in its framing in a sensitive geo-political context, marked by unprecedented pressures on the provision of consumption needs, oil price increases and inflation. For the analysed countries, the risk function is an indicator of the need to change public policies in order to reduce energy dependence and can be extrapolated to all countries in the world using the econometric function proposed in the Methodology section.

The study conducted by the authors is new and because it focuses on a highly topical issue—energy dependence—and provides a model for assessing energy risk by quantifying energy dependence in relation to the sector's primary indicators (production and imports) and in relation to economic growth as a decelerator of dependence.

The study is useful to decision-makers for the implementation of energy security policies and the projection of medium-term energy strategies.

The limitations of the study lie in the relatively limited number of indicators analysed, and the authors propose to extend the analysis in future research and to make a more detailed projection of the risk of energy dependency.
